# A comparative study of topological entropy characterization and graph energy prediction for Marta variants of covalent organic frameworks

**DOI:** 10.3389/fchem.2024.1511678

**Published:** 2024-12-20

**Authors:** Zahid Raza, Micheal Arockiaraj, Aravindan Maaran, Arul Jeya Shalini

**Affiliations:** ^1^ Department of Mathematics, College of Sciences, University of Sharjah, Sharjah, United Arab Emirates; ^2^ Department of Mathematics, Loyola College, Chennai, India; ^3^ Department of Mathematics, Loyola College, University of Madras, Chennai, India; ^4^ Department of Mathematics, Women’s Christian College, Chennai, India

**Keywords:** hexabenzocoronenes, covalent organic frameworks, vertex degree indices, entropies, graph energy

## Abstract

Covalent organic frameworks are a novel class of porous polymers, notable for their crystalline structure, intricate frameworks, defined pore sizes, and capacity for structural design, synthetic control, and functional customization. This paper provides a comprehensive analysis of graph entropies and hybrid topological descriptors, derived from geometric, harmonic, and Zagreb indices. These descriptors are applied to study two variations of Marta covalent organic frameworks based on contorted hexabenzocoronenes. We also conduct a comparative analysis using scaled entropies, offering refined tools for assessing the intrinsic topologies of these networks. Additionally, these hybrid descriptors are used to develop statistical models for predicting graph energy in higher-dimensional Marta-COFs.

## 1 Introduction

Reticular chemistry connects organic building blocks through strong covalent bonds, which have the capacity to regulate the pore sizes of frameworks by preserving their fundamental topology and varying the lengths of organic linkers, thus paving the way for the emergence of multiple classes of crystalline porous materials (Yaghi, 2016; Yaghi, 2019). Reticulated materials can be classified as metal organic frameworks (MOFs), created by the combination of organic linkers and metal atoms, and covalent organic frameworks (COFs), composed only of organic linkers (Gropp et al., 2020). COFs have drawn particular attention from researchers due to their regular pattern of organic building blocks, which allows for the creation of crystalline structures with extensive surface areas, stability, and customizable pores (El-Kaderi et al., 2007). COFs possess potential applicatiions in separation (Fan et al., 2023), luminescence (Haug et al., 2020), biomedicine (Shi et al., 2023), energy conversion (Sun et al., 2023), environmental remediation (Hou et al., 2023), seawater desalination (Jrad et al., 2023), photocatalysis (Gong et al., 2023), and electrocatalysis (Zhang et al., 2021). The COFs have predetermined structures based on their building blocks, allowing for highly ordered geometries (Huang et al., 2016; Chen et al., 2014). Their covalently crystalline structure gives them advantages over other porous materials such as molecular sieves, MOFs and zeolites (Yang et al., 2019; Jiao et al., 2019; Algieri and Drioli, 2021).

Covalent bonds within COFs can arise from a diverse range of functional groups. The methods for forming these bonds can be broadly classified into several categories, including boroxine-linked, boronate ester-linked, triazine-linked, imine-linked, hydrazone-linked, β -ketoenamine-linked, azine-linked, imide-linked, carbon-carbon linked, and others (Wang et al., 2020; Geng et al., 2020). Boronate ester based COFs represent a category of crystalline, porous polymers characterized by layer-stacked structures, which are formed through reversible covalent interactions between boronic acid and catechol. The initial reported methods for COF formation involved the self-condensation of boronic acids into boroxine rings and the co-condensation of boronic acids with catechols to form boronic esters. This bond type stands out as one of the most frequently observed COF formation, with COF-5 being an early example falling within this category (Cot̂é et al., 2005; Li et al., 2018). Since then the varieties of COFs featuring boron, have gained significant attention primarily due to their exceptional thermal stability (Kuhn et al., 2008).

**FIGURE 1:**
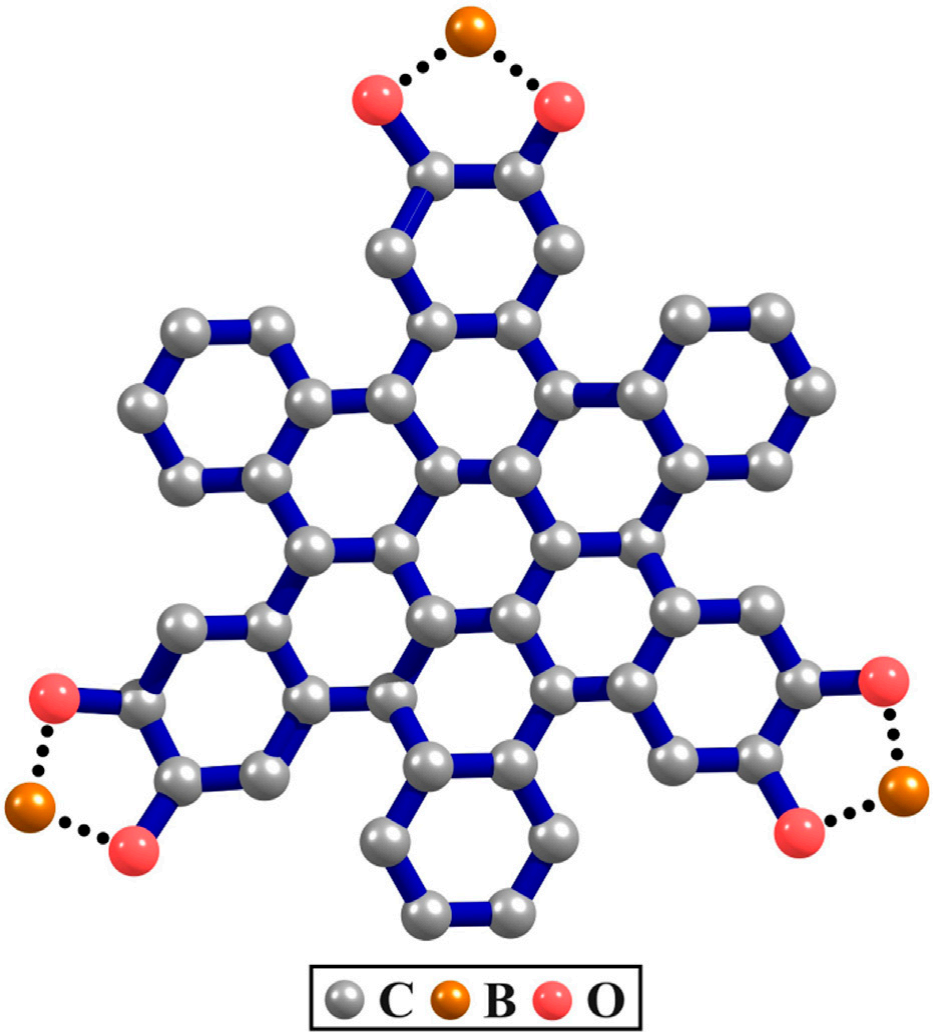
Contorted hexabenzocoronene (c-HBC).

**FIGURE 2:**
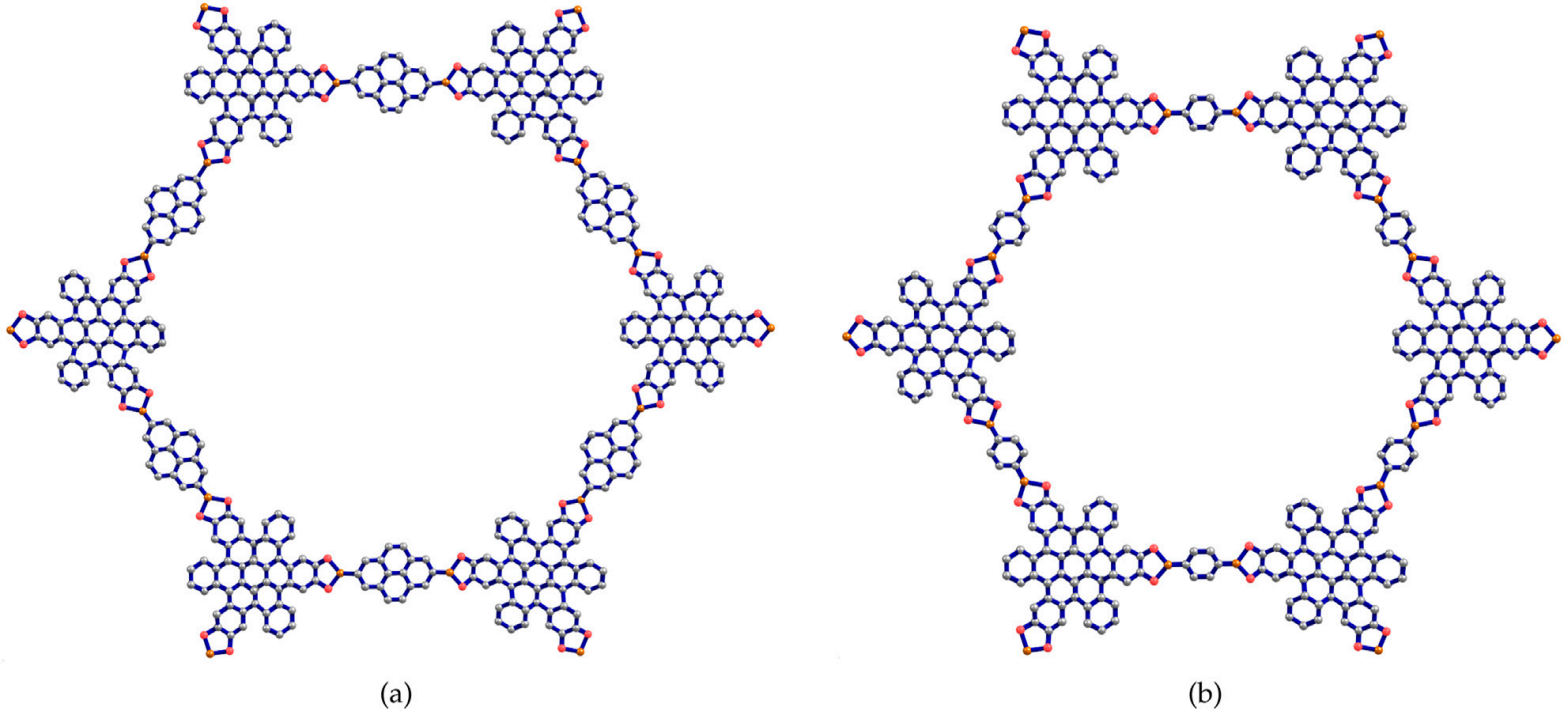
The unit cell structures of **(A)** Marta-COF-1 **(B)** Marta-COF-2.

The COFs considered in this study are made up of polycyclic aromatic hydrocarbons (PAHs) with contorted hexabenzocoronene (c-HBC) serving as the core component for constructing the two Marta-COFs. The c-HBC adopts a doubly-concave structure, which sets it apart from the planar hexabenzocoronene. Its formation occurs when the aromatic core of HBC is distorted away from planarity due to steric congestion in its proximal carbon atoms. Structurally, c-HBC is the building block composed of six benzene rings attached to the periphery of a coronene molecule (Sepúlveda et al., 2017; Kim et al., 2022). The c-HBC unit, as shown in Figure 1, when copolymerized with pyrene-2,7-diboronic acid (PDBA), results in the formation of a highly crystalline two-dimensional COF known as Marta-COF-1 (Abadía et al., 2019). Notably, the c-HBC nodes and the PDBA display substantial π -areas and extensive π -stacking within the resulting COF. In response to this, a comparable COF labeled Marta-COF-2 has been created, synthesized and investigated with the substitution of pyrene-2,7-diboronic acid by benzene-1,4-diboronic acid (BDBA) (Abadía et al., 2021). As depicted in Figure 2, graphical diagram representations of the two COF frameworks, Marta-COF-1 and Marta-COF-2, are illustrated to highlight their distinctive arrangements.

The two variations of highly crystalline Marta COFs can be evaluated through quantitative parameters called the topological descriptors that convert various structural attributes of the frameworks into measurable quantities. These quantifying functionals are essential for representing the molecular frameworks and are useful for QSPR and QSAR analyses (Jafari et al., 2024; Patil et al., 2024; Nath et al., 2023; Hayat et al., 2023). The incorporation of topological descriptors and graph-derived metrics in QSAR/QSPR studies has been extensively used in the domain of computational and material sciences. This amalgamation has provided robust tools for predicting structural behaviors and designing new materials with desired properties or functionalities. These approaches enable researchers to explore numerous applications, including drug discovery, material optimization, and the development of materials that can be tailored for specific applications or objectives (Balasubramanian and Saxena, 2021; Balasubramanian, 2022; Arockiaraj et al., 2023a; Hasani and Ghods, 2024; Abubakar et al., 2024; Meharban et al., 2024; Ullah et al., 2024; Shanmukha et al., 2023a; Gnanaraja et al., 2023; Zhang et al., 2023; Hassan et al., 2024).

The graph entropy measure enables the evaluation of the inherent complexity and diversity of COFs. This measure provides valuable insights into the arrangement and functioning of COF structures by associating fundamental graph components with appropriate weights (Junias and Clement, 2023; Arockiaraj et al., 2024a; Chu et al., 2023; Roy et al., 2023; Zhao et al., 2023; Lal et al., 2024). The applications of graph entropy continue to expand its relevance and significance across diverse domains due to its adaptable nature that surpasses disciplinary boundaries and facilitates the analysis of complex systems (Arockiaraj et al., 2023b; Junias et al., 2024; Huang et al., 2024). In recent years, there has been significant interest in the computation of topological expressions and entropies for COFs (Yang et al., 2024; Arockiaraj et al., 2023c; Augustine and Roy, 2022; Shanmukha et al., 2023b; Arockiaraj et al., 2024b). In this study, we provide hybrid topological characterizations and entropies for two variations of Marta COFs and conduct a comparative analysis of the bond-wise entropy of these frameworks. Furthermore, we construct regression models to predict the graph energy of these frameworks based on the calculated topological indices.

## 2 Computational methods

We consider the Marta-COF as a molecular graph where the sets V(Marta-COF) and E(Marta-COF) represent the atoms and bonds respectively. Our mathematical computation involves deriving topological descriptors and entropies, incorporating hybrid descriptors based on vertex degree and degree-sum parameters. The number of bonds incident to a vertex p∈V(Marta-COF) is denoted as $d_{\text{Marta}-\text{COF}}\left(p\right)$ which represents the degree of a vertex p . Additionally, the total sum of the degrees of all neighbors of vertex p is denoted as $s_{\text{Marta}-\text{COF}}\left(p\right)$ which is defined as the degree-sum of vertex p . That is, $s_{\text{Marta}-\text{COF}}\left(p\right)=\sum_{q\in N_{\text{Marta}-\text{COF}}\left(p\right)}d_{\text{Marta}-\text{COF}}\left(q\right)$ in which we used $N_{\text{Marta}-\text{COF}}\left(p\right)=\left\{q\in V\left(\text{Marta}-\text{COF}\right)\;|\;pq\in E\left(\text{Marta}-\text{COF}\right)\right\}$ . Let $d\left(r,x\right)=|\left\{mn\in E\left(\text{Marta}-\text{COF}\right)\;:r=\;d_{\text{Marta}-\text{COF}}\left(m\right)\;\text{and}\;x=d_{\text{Marta}-\text{COF}}\left(n\right)\right\}|$ and $s\left(r,x\right)=|\left\{mn\in E\left(\text{Marta}-\text{COF}\right)\;:r=\;s_{\text{Marta}-\text{COF}}\left(m\right)\;\text{and}\;x=s_{\text{Marta}-\text{COF}}\left(n\right)\right\}|$ . The total number of edges within Marta-COFs is classified into distinct edge classes based on symmetrical representations related to d(r,x) and s(r,x) . These edge classes are labeled as D (Marta-COF) and S (Marta-COF), respectively.

We now define the additive and multiplicative versions of topological descriptors related to the degree and degree-sum parameters of Marta-COF, involving the index function ξ , as follows (Hakeem et al., 2023; Paul et al., 2023; Arockiaraj et al., 2022; Mondal et al., 2022; Arockiaraj et al., 2023d; Ramane et al., 2021; Yu et al., 2023; Zaman et al., 2023; Hassan et al., 2024):

$$\xi^{d}\left(Marta-COF\right)=\underset{d\left(r,x\right)\in D\left(\text{Marta}-\text{COF}\right)}{\sum }d\left(r,x\right)\xi \left(r,x\right)$$

$$\xi^{d^{*}}\left(Marta-COF\right)=\underset{d\left(r,x\right)\in D\left(\text{Marta}-\text{COF}\right)}{\prod }d\left(r,x\right)\xi \left(r,x\right)$$

$$\xi^{s}\left(Marta-COF\right)=\underset{s\left(r,x\right)\in S\left(\text{Marta}-\text{COF}\right)}{\sum }s\left(r,x\right)\xi \left(r,x\right)$$

$$\xi^{s^{*}}\left(Marta-COF\right)=\underset{s\left(r,x\right)\in S\left(\text{Marta}-\text{COF}\right)}{\prod }s\left(r,x\right)\xi \left(r,x\right)$$

When the index function ξ is raised to its own power, the resulting versions of topological descriptors can take the following forms:

$$\xi^{dp}\left(Marta-COF\right)=\underset{d\left(r,x\right)\in D\left(\text{Marta}-\text{COF}\right)}{\sum }d\left(r,x\right)\xi {\left(r,x\right)}^{\xi \left(r,x\right)}$$

$$\xi^{dp^{*}}\left(Marta-COF\right)=\underset{d\left(r,x\right)\in D\left(\text{Marta}-\text{COF}\right)}{\prod }d\left(r,x\right)\xi {\left(r,x\right)}^{\xi \left(r,x\right)}$$

$$\xi^{sp}\left(Marta-COF\right)=\underset{s\left(r,x\right)\in S\left(\text{Marta}-\text{COF}\right)}{\sum }s\left(r,x\right)\xi {\left(r,x\right)}^{\xi \left(r,x\right)}$$

$$\xi^{sp^{*}}\left(Marta-COF\right)=\underset{s\left(r,x\right)\in S\left(\text{Marta}-\text{COF}\right)}{\prod }s\left(r,x\right)\xi {\left(r,x\right)}^{\xi \left(r,x\right)}$$

The index functions ξ(r,x) are considered in our study, as stated below (Arockiaraj et al., 2024a; Arockiaraj et al., 2024b; Arockiaraj et al., 2023e; Arockiaraj et al., 2024c).

• BM(r,x)=r+x+rx (Bi − Zagreb)

• $TM\left(r,x\right)=r^{2}+x^{2}+rx$ (Tri − Zagreb)

• $GH\left(r,x\right)=\frac{\sqrt{rx}\left(r+x\right)}{2}$ (Geometric − Harmonic)

• $GBM\left(r,x\right)=\frac{\sqrt{rx}}{r+x+rx}$ (Geometric − Bi-Zagreb)

• $GTM\left(r,x\right)=\frac{\sqrt{rx}}{r^{2}+x^{2}+rx}$ (Geometric − Tri-Zagreb)

• $HG\left(r,x\right)=\frac{2}{\sqrt{rx}\left(r+x\right)}$ (Harmonic − Geometric)

• $HBM\left(r,x\right)=\frac{2}{\left(r+x+rx\right)\left(r+x\right)}$ (Harmonic − Bi-Zagreb)

• $HTM\left(r,x\right)=\frac{2}{\left(r^{2}+x^{2}+rx\right)\left(r+x\right)}$ (Harmonic − Tri-Zagreb)

• $BMG\left(r,x\right)=\frac{\left(r+x+rx\right)}{\sqrt{rx}}$ (Bi-Zagreb − Geometric)

• $BMH\left(r,x\right)=\frac{\left(r+x+rx\right)\left(r+x\right)}{2}$ (Bi-Zagreb − Harmonic)

• $TMG\left(r,x\right)=\frac{r^{2}+x^{2}+rx}{\sqrt{rx}}$ (Tri-Zagreb − Geometric)

• $TMH\left(r,x\right)=\frac{\left(r^{2}+x^{2}+rx\right)\left(r+x\right)}{2}$ (Tri-Zagreb − Harmonic)

These index functions, combined with the edge classes based on d(r,x) and s(r,x) , lead to the formation of topological descriptors. However, the representative element in the edge classes does not account for the specific types of atoms involved at their terminal points. Since three types of atoms are present in Marta covalent organic frameworks, which constitute the basis for Marta, it is important to distinguish between the atoms. Therefore, we involve weight functions that consider both the atoms and bonds, thereby enhancing the partitions based on d(r,x) and s(r,x) . The weight function for atoms will be denoted by the symbol Φ , while Γ will represent the weight function for bonds. Particularly, $\Phi_{\text{B}}$ represents the weight assigned to atom B, while $\Gamma_{\text{BC}}$ denotes the weight function corresponding to the bond B − C. As a result, the edge classification of Marta-COFs will undergo additional refinement through the utilization of the bond weight function.

In employing Shannon’s entropy method, defining a structural information function on the bonds of Marta-COFs is necessary. In our study, we adopt the index function ξ derived from degree or degree-sum parameters of Marta-COFs corresponding to the structural information function. The entropy of Marta-COF structures using the structural information function ξ is defined on $E\left(\text{Marta}-\text{COF}\right)=\left\{c_{1},c_{2},\ldots ,c_{m}\right\}$ and takes the following form.

$$\begin{array}{lll} I_{\xi }\left(Marta-COF\right) & =-\overset{m}{\underset{x=1}{\sum }}\frac{\xi \left(c_{x}\right)}{\overset{m}{\underset{z=1}{\sum }}\xi \left(c_{z}\right)}\log\left(\frac{\xi \left(c_{x}\right)}{\overset{m}{\underset{z=1}{\sum }}\xi \left(c_{z}\right)}\right) & \\ & =\log\left(\overset{m}{\underset{x=1}{\sum }}\xi \left(c_{x}\right)\right)-\frac{1}{\overset{m}{\underset{x=1}{\sum }}\xi \left(c_{x}\right)}\log\left(\overset{m}{\underset{c=1}{\prod }}\xi {\left(c_{x}\right)}^{\xi \left(c_{x}\right)}\right) & \end{array}$$

In a series of papers (Arockiaraj et al., 2023c; Mushtaq et al., 2022; Raza et al., 2023), the significance and implications of substituting the multiplicative factor have been comprehensively explored concerning the scalar multiplicative index. This leads to the formulation of the modified version of entropy as presented below.

$$I_{\xi }\left(Marta-COF\right)=\log\left(\xi \left(Marta-COF\right)\right)-\frac{1}{\xi \left(Marta-COF\right)}\log\left(\xi^{p*}\left(Marta-COF\right)\right)$$

## 3 Results and discussion

In this section, the two types of Marta-COFs are analyzed, and their structural properties are compared using topological descriptors and entropies. We consider the geometrical configuration of bi-trapezium (BT) shaped arrangements of Marta-COFs, which yield diverse configurations of Marta-COF layers. These Marta-COF structures are constructed using the unit cells as shown in Figure 2, which are the fundamental building blocks.

**FIGURE 3:**
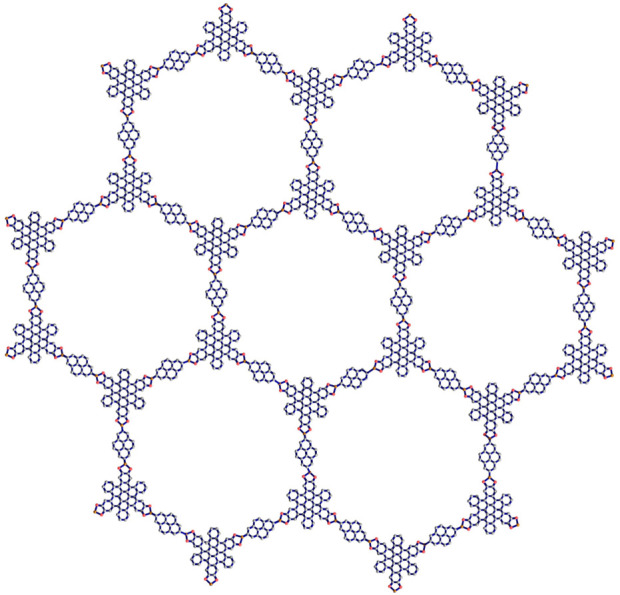
Hexagonal Marta-COF-1 with dimension 2.

**FIGURE 4:**
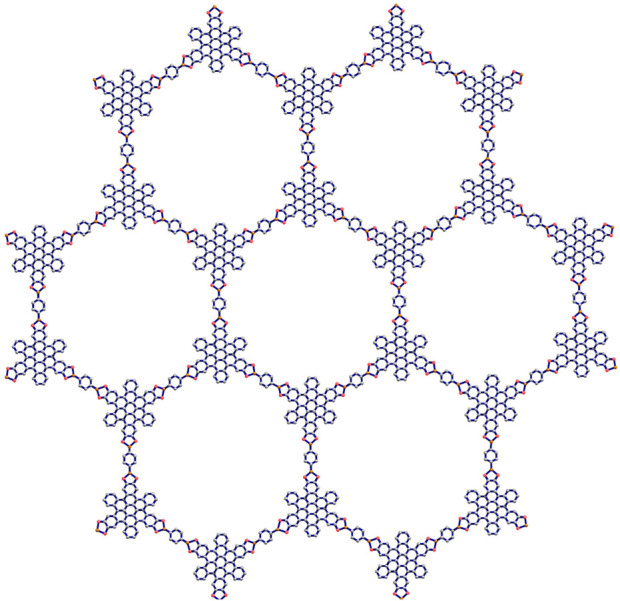
Hexagonal Marta-COF-2 with dimension 2.

The Marta-COF-BT (t,u) geometric formation is achieved by arranging t units linearly to form the base and u units to form the non-parallel sides, subject to the conditions t≥2 and u≤t . By fixing t=2u−1 and t=u respectively, the hexagonal and parallelogram geometries are extracted from the BT configurations which are denoted by Marta-COF-H (u) and Marta-COF-P (u,u) . The linear chain of Marta-COFs is derived by setting t=1 and is represented as Marta-COF-L (u) . The representations of hexagonal structures for two variations of Marta-COFs are depicted in Figures 3, 4.

Furthermore, the covalent organic framework Marta-COF-1-BT (t,u) is composed of $324tu-162u^{2}-16t+308u-16$ vertices and $414tu-207u^{2}-21t+393u-21$ edges, while Marta-COF-2-BT (t,u) comprises $264tu-132u^{2}-6t+258u-6$ vertices and $336tu-168u^{2}-8t+328u-8$ edges. We have computed diverse molecular descriptors of Marta COFs by calculating degree and degree-sum parameters and the distribution of bonds are shown in Tables 1, 2. The explicit mathematical expressions for these descriptors in Marta-COFs are derived by assigning unit weights to atoms and bonds.

**TABLE 1:** Bond partitioning of Marta-COF-1-BT (t,u) and Marta-COF-2-BT (t,u) according to degree classes.

Bond S − T	$\left(d_{\text{Marta}-\text{COF}}\left(\text{S}\right),d_{\text{Marta}-\text{COF}}\left(\text{T}\right)\right)$	Number of degree bonds	
		Marta-COF-1-BT (t,u)	Marta-COF-2-BT (t,u)
B − O	$\left(2\Phi_{\text{O}},\Phi_{\text{B}}+\Phi_{\text{C}}\right)$	4t+4u+4	4t+4u+4
	$\left(2\Phi_{\text{O}}+\Phi_{\text{C}},\Phi_{\text{B}}+\Phi_{\text{C}}\right)$	$24tu-12u^{2}-4t+20u-4$	$24tu-12u^{2}-4t+20u-4$
O − C	$\left(\Phi_{\text{B}}+\Phi_{\text{C}},2\Phi_{\text{C}}+\Phi_{\text{O}}\right)$	$24tu-12u^{2}+24u$	$24tu-12u^{2}+24u$
C − C	$\left(3\Phi_{\text{C}},3\Phi_{\text{C}}\right)$	$150tu-75u^{2}-5t+145u-5$	$120tu-60u^{2}+120u$
	$\left(2\Phi_{\text{C}},3\Phi_{\text{C}}\right)$	$120tu-60u^{2}-12t+108u-12$	$72tu-36u^{2}-4t+68u-4$
	$\left(2\Phi_{\text{C}}+\Phi_{\text{O}},2\Phi_{\text{C}}\right)$	$24tu-12u^{2}+24u$	$24tu-12u^{2}+24u$
	$\left(2\Phi_{\text{C}}+\Phi_{\text{O}},2\Phi_{\text{C}}+\Phi_{\text{O}}\right)$	$12tu-6u^{2}+12u$	$12tu-6u^{2}+12u$
	$\left(2\Phi_{\text{C}},2\Phi_{\text{C}}\right)$	$48tu-24u^{2}-2t+46u-2$	$48tu-24u^{2}-2t+46u-2$
B − C	$\left(2\Phi_{\text{O}}+\Phi_{\text{C}},2\Phi_{\text{C}}+\Phi_{\text{B}}\right)$	$12tu-6u^{2}-2t+10u-2$	$12tu-6u^{2}-2t+10u-2$

**TABLE 2:** Bond partitioning of Marta-COF-1-BT (t,u) and Marta-COF-2-BT (t,u) according to degree-sum classes.

Bond S − T	$\left(s_{\text{Marta}-\text{COF}}\left(\text{S}\right),s_{\text{Marta}-\text{COF}}\left(\text{T}\right)\right)$	Number of degree-sum bonds	
		Marta-COF-1-BT (t,u)	Marta-COF-2-BT (t,u)
B − O	$\left(4\Phi_{\text{O}},3\Phi_{\text{C}}+2\Phi_{\text{B}}\right)$	4t+4u+4	4t+4u+4
	$\left(4\Phi_{\text{O}}+3\Phi_{\text{C}},3\Phi_{\text{B}}+3\Phi_{\text{C}}\right)$	$24tu-12u^{2}-4t+20u-4$	$24tu-12u^{2}-4t+20u-4$
O − C	$\left(2\Phi_{\text{B}}+3\Phi_{\text{C}},5\Phi_{\text{C}}+2\Phi_{\text{O}}\right)$	4t+4u+4	4t+4u+4
	$\left(3\Phi_{\text{B}}+3\Phi_{\text{C}},5\Phi_{\text{C}}+2\Phi_{\text{O}}\right)$	$24tu-12u^{2}-4t+20u-4$	$24tu-12u^{2}-4t+20u-4$
C − C	$\left(8\Phi_{\text{C}},8\Phi_{\text{C}}\right)$	$24tu-12u^{2}+24u$	$24tu-12u^{2}+24u$
	$\left(9\Phi_{\text{C}},9\Phi_{\text{C}}\right)$	$54tu-27u^{2}-t+53u-1$	$48tu-24u^{2}+48u$
	$\left(2\Phi_{\text{O}}+5\Phi_{\text{C}},2\Phi_{\text{O}}+5\Phi_{\text{C}}\right)$	$12tu-6u^{2}+12u$	$12tu-6u^{2}+12u$
	$\left(8\Phi_{\text{C}},9\Phi_{\text{C}}\right)$	$48tu-24u^{2}+48u$	$48tu-24u^{2}+48u$
	$\left(6\Phi_{\text{C}},8\Phi_{\text{C}}\right)$	$24tu-12u^{2}+24u$	$24tu-12u^{2}+24u$
	$\left(5\Phi_{\text{C}},8\Phi_{\text{C}}\right)$	$24tu-12u^{2}+24u$	$24tu-12u^{2}+24u$
	$\left(4\Phi_{\text{C}},5\Phi_{\text{C}}\right)$	$24tu-12u^{2}+24u$	$24tu-12u^{2}+24u$
	$\left(4\Phi_{\text{C}},4\Phi_{\text{C}}\right)$	$12tu-6u^{2}+12u$	$12tu-6u^{2}+12u$
	$\left(6\Phi_{\text{C}},7\Phi_{\text{C}}\right)$	$72tu-36u^{2}-8t+64u-8$	$24tu-12u^{2}+24u$
	$\left(5\Phi_{\text{C}},5\Phi_{\text{C}}\right)$	$12tu-6u^{2}-2t+10u-2$	$12tu-6u^{2}-2t+10u-2$
	$\left(5\Phi_{\text{C}},7\Phi_{\text{C}}\right)$	$24tu-12u^{2}-4t+20u-4$	$24tu-12u^{2}-4t+20u-4$
	$\left(7\Phi_{\text{C}},9\Phi_{\text{C}}\right)$	$24tu-12u^{2}-4t+20u-4$	−
B − C	$\left(4\Phi_{\text{O}}+3\Phi_{\text{C}},4\Phi_{\text{C}}+3\Phi_{\text{B}}\right)$	$12tu-6u^{2}-2t+10u-2$	$12tu-6u^{2}-2t+10u-2$

The degree based descriptors for Marta-COF-1-BT (t,u) are obtained for ξ using the following equation.

$$\begin{array}{ll} \xi^{d}\left(Marta-COF-1-BT\left(\text{t,u}\right)\right) & =\left(4t+4u+4\right)\Gamma_{\text{BO}}\xi \left(2\Phi_{\text{O}},\Phi_{\text{B}}+\Phi_{\text{C}}\right)+\left(24tu-12u^{2}-4t+20u-4\right)\Gamma_{\text{BO}}\\ & \;\;\;\times \xi \left(2\Phi_{\text{O}}+\Phi_{\text{C}},\Phi_{\text{B}}+\Phi_{\text{C}}\right)+\left(12tu-6u^{2}-2t+10u-2\right)\Gamma_{\text{BC}}\xi \left(2\Phi_{\text{O}}+\Phi_{\text{C}},2\Phi_{\text{C}}+\Phi_{\text{B}}\right)\\ & \;\;\;+\;\left(24tu-12u^{2}+24u\right)\Gamma_{\text{OC}}\xi \left(\Phi_{\text{B}}+\Phi_{\text{C}},2\Phi_{\text{C}}+\Phi_{\text{O}}\right)+\left(150tu-75u^{2}-5t+145u-5\right)\\ & \;\;\;\times \Gamma_{\text{CC}}\xi \left(3\Phi_{\text{C}},3\Phi_{\text{C}}\right)+\left(24tu-12u^{2}+24u\right)\Gamma_{\text{CC}}\xi \left(2\Phi_{\text{C}}+\Phi_{\text{O}},2\Phi_{\text{C}}\right)+\left(12tu-6u^{2}+12u\right)\\ & \;\;\;\times \Gamma_{\text{CC}}\xi \left(2\Phi_{\text{C}}+\Phi_{\text{O}},2\Phi_{\text{C}}+\Phi_{\text{O}}\right)+\left(120tu-60u^{2}-12t+108u-12\right)\Gamma_{\text{CC}}\\ & \;\;\;\times \xi \left(2\Phi_{\text{C}},3\Phi_{\text{C}}\right)+\left(48tu-24u^{2}-2t+46u-2\right)\Gamma_{\text{CC}}\xi \left(2\Phi_{\text{C}},2\Phi_{\text{C}}\right) \end{array}$$

In computing the degree-sum descriptors of Marta-COF-1-BT (t,u) , we use

$$\begin{array}{ll} \xi^{s}\left(Marta-COF-1-BT\left(\text{t,u}\right)\right) & =\left(4t+4u+4\right)\Gamma_{\text{BO}}\xi \left(4\Phi_{\text{O}},3\Phi_{\text{C}}+2\Phi_{\text{B}}\right)+\left(24tu-12u^{2}-4t+20u-4\right)\\ & \;\;\;\times \Gamma_{\text{BO}}\xi \left(4\Phi_{\text{O}}+3\Phi_{\text{C}},3\Phi_{\text{B}}+3\Phi_{\text{C}}\right)+\left(24tu-12u^{2}-4t+20u-4\right)\\ & \;\;\;\times \Gamma_{\text{OC}}\xi \left(3\Phi_{\text{B}}+3\Phi_{\text{C}},5\Phi_{\text{C}}+2\Phi_{\text{O}}\right)+\left(4t+4u+4\right)\Gamma_{\text{OC}}\xi \left(2\Phi_{\text{B}}+3\Phi_{\text{C}},5\Phi_{\text{C}}+2\Phi_{\text{O}}\right)\\ & \;\;\;+\;\left(12tu-6u^{2}+12u\right)\Gamma_{\text{CC}}\xi \left(2\Phi_{\text{O}}+5\Phi_{\text{C}},2\Phi_{\text{O}}+5\Phi_{\text{C}}\right)+\left(48tu-24u^{2}+48u\right)\\ & \;\;\;\times \Gamma_{\text{CC}}\xi \left(8\Phi_{\text{C}},9\Phi_{\text{C}}\right)+\left(24tu-12u^{2}+24u\right)\Gamma_{\text{CC}}\xi \left(8\Phi_{\text{C}},8\Phi_{\text{C}}\right)+\left(24tu-12u^{2}+24u\right)\\ & \;\;\;\times \Gamma_{\text{CC}}\xi \left(6\Phi_{\text{C}},8\Phi_{\text{C}}\right)+\left(24tu-12u^{2}+24u\right)\Gamma_{\text{CC}}\xi \left(5\Phi_{\text{C}},8\Phi_{\text{C}}\right)+\left(24tu-12u^{2}+24u\right)\\ & \;\;\;\times \Gamma_{\text{CC}}\xi \left(4\Phi_{\text{C}},5\Phi_{\text{C}}\right)+\left(54tu-27u^{2}-t+53u-1\right)\Gamma_{\text{CC}}\xi \left(9\Phi_{\text{C}},9\Phi_{\text{C}}\right)+\left(12tu-6u^{2}+12u\right)\\ & \;\;\;\times \Gamma_{\text{CC}}\xi \left(4\Phi_{\text{C}},4\Phi_{\text{C}}\right)+\left(72tu-36u^{2}-8t+64u-8\right)\Gamma_{\text{CC}}\xi \left(6\Phi_{\text{C}},7\Phi_{\text{C}}\right)\\ & \;\;\;+\;\left(12tu-6u^{2}-2t+10u-2\right)\Gamma_{\text{CC}}\xi \left(5\Phi_{\text{C}},5\Phi_{\text{C}}\right)+\left(24tu-12u^{2}-4t+20u-4\right)\\ & \;\;\;\times \Gamma_{\text{CC}}\xi \left(5\Phi_{\text{C}},7\Phi_{\text{C}}\right)+\left(24tu-12u^{2}-4t+20u-4\right)\Gamma_{\text{CC}}\xi \left(7\Phi_{\text{C}},9\Phi_{\text{C}}\right)\\ & \;\;\;+\;\left(12tu-6u^{2}-2t+10u-2\right)\Gamma_{\text{BC}}\xi \left(4\Phi_{\text{O}}+3\Phi_{\text{C}},4\Phi_{\text{C}}+3\Phi_{\text{B}}\right). \end{array}$$

The resulting outcomes are given in the form, $\xi^{\#}\;\left(\text{Marta}-\text{COF}\right)\;=\;\left\{\xi^{d}\left(\text{Marta}-\text{COF}\right),\xi^{s}\left(\text{Marta}-\text{COF}\right)\right\}$ .

Result 1 The quantitative expressions for Marta-COF-1-BT (t,u) are given by

1. $\begin{array}{cc} & GH^{\#}\left(Marta-COF-1-BT\left(t,u\right)\right)=\left\{\left(tu\left(480\sqrt{6}+1758\right)-u^{2}\left(240\sqrt{6}+879\right)-t\left(40\sqrt{6}+55\right)\right.\right.\\ & \left.+u\left(440\sqrt{6}+1703\right)-40\sqrt{6}-55\right),\left(tu\left(672\sqrt{3}+216\sqrt{5}+\sqrt{2}\left(312\sqrt{5}+2448\right)+\sqrt{7}\left(780\sqrt{6}+144\sqrt{5}\right.\right.\right.\\ & \left.\left.+576\right)+7578\right)-u^{2}\left(336\sqrt{3}+108\sqrt{5}+\sqrt{2}\left(156\sqrt{5}+1224\right)+\sqrt{7}\left(390\sqrt{6}+72\sqrt{5}+288\right)+3789\right)\\ & -t\left(\sqrt{7}\left(104\sqrt{6}+96\right)-36\sqrt{5}+229\right)+u\left(672\sqrt{3}+252\sqrt{5}+\sqrt{2}\left(312\sqrt{5}+2448\right)+\sqrt{7}\left(676\sqrt{6}\right.\right.\\ & \left.\left.\left.\left.+144\sqrt{5}+480\right)+7349\right)+36\sqrt{5}-\sqrt{7}\left(104\sqrt{6}+96\right)-229\right)\right\} \end{array}$

2. $\begin{array}{cc} & GBM^{\#}\left(Marta-COF-1-BT\left(t,u\right)\right)=\left\{\left(\left(tu\left(1920\sqrt{6}+5148\right)-u^{2}\left(960\sqrt{6}+2574\right)-t\left(160\sqrt{6}\right.\right.\right.\right.\\ & \left.\left.\left.+99\right)+u\left(1760\sqrt{6}+5049\right)-160\sqrt{6}-99\right)/110\right),\left(\left(tu\left(\sqrt{2}\left(49410485807760\sqrt{5}+176545331313120\right)\right.\right.\right.\\ & +\sqrt{7}\left(119034352173240\sqrt{6}+27859103700120\sqrt{5}+49723210401480\right)+84475991864880\sqrt{3}+90301922338320\sqrt{5}\\ & \left.+746893324033044\right)-u^{2}\left(\sqrt{7}\left(59517176086620\sqrt{6}+13929551850060\sqrt{5}+24861605200740\right)+42237995932440\sqrt{3}\right.\\ & \left.+\sqrt{5}45150961169160+\sqrt{2}\left(24705242903880\sqrt{5}+88272665656560\right)+373446662016522\right)-t\left(\sqrt{7}\left(15871246956432\sqrt{6}\right.\right.\\ & \left.\left.+8287201733580\right)-15050320389720\sqrt{5}+32671465708925\right)+u\left(\sqrt{2}\left(176545331313120+49410485807760\sqrt{5}\right)\right.\\ & +84475991864880\sqrt{3}+105352242728040\sqrt{5}+\sqrt{7}\left(103163105216808\sqrt{6}+27859103700120\sqrt{5}+41436008667900\right)\\ & \left.\left.\left.\left.+714221858324119\right)-\sqrt{7}\left(15871246956432\sqrt{6}+8287201733580\right)+15050320389720\sqrt{5}-32671465708925\right)/54557411412735\right)\right\} \end{array}$

3. $\begin{array}{cc} & GTM^{\#}\left(Marta-COF-1-BT\left(t,u\right)\right)=\left\{\left(tu\left(1728\sqrt{6}+4674\right)-u^{2}\left(864\sqrt{6}+2337\right)-t\left(144\sqrt{6}+76\right)\right.\right.\\ & \left.+u\left(1584\sqrt{6}+4598\right)-144\sqrt{6}-76/171\right),\left(\left(tu\left(\sqrt{7}\left(7177258197559800\sqrt{6}+1672498699247880\sqrt{5}+2833715412715320\right)\right.\right.\right.\\ & +4927090762649160\sqrt{3}+5977126498951440\sqrt{5}+\sqrt{2}\left(2826393150666960\sqrt{5}+10081236399153120\right)\\ & \left.+45141536320652304\right)-u^{2}\left(\sqrt{7}\left(3588629098779900\sqrt{6}+836249349623940\sqrt{5}+1416857706357660\right)\right.\\ & \left.+2463545381324580\sqrt{3}+2988563249475720\sqrt{5}+\sqrt{2}\left(1413196575333480\sqrt{5}+5040618199576560\right)+22570768160326152\right)\\ & -t\left(\sqrt{7}\left(956967759674640\sqrt{6}+472285902119220\right)+2017543735128869-\sqrt{5}996187749825240\right)\\ & +u\left(\sqrt{7}\left(6220290437885160\sqrt{6}+1672498699247880\sqrt{5}+2361429510596100\right)+4927090762649160\sqrt{3}\right.\\ & \left.+6973314248776680\sqrt{5}+\sqrt{2}\left(2826393150666960\sqrt{5}+10081236399153120\right)+43123992585523435\right)+996187749825240\sqrt{5}\\ & \left.\left.\left.-\sqrt{7}\left(956967759674640\sqrt{6}+472285902119220\right)-2017543735128869\right)/7595931592417455\right)\right\} \end{array}$

4. $\begin{array}{cc} & HG^{\#}\left(Marta-COF-1-BT\left(t,u\right)\right)=\left\{\left(\left(tu\left(1152\sqrt{6}+2820\right)-u^{2}\left(576\sqrt{6}+1410\right)-t\left(96\sqrt{6}\right.\right.\right.\right.\\ & \left.\left.\left.+25\right)+u\left(1056\sqrt{6}+2795\right)-96\sqrt{6}-25\right)/90\right),\left(\left(tu\left(\sqrt{7}\left(154224000\sqrt{6}+40098240\sqrt{5}+50122800\right)+100245600\sqrt{3}\right.\right.\right.\\ & \left.+187125120\sqrt{5}+\sqrt{2}\left(64774080\sqrt{5}+165110400\right)+968885658\right)-u^{2}\left(\sqrt{7}\left(77112000\sqrt{6}+20049120\sqrt{5}\right.\right.\\ & \left.\left.+25061400\right)+50122800\sqrt{3}+93562560\sqrt{5}+\sqrt{2}\left(32387040\sqrt{5}+82555200\right)+484442829\right)-t\left(\sqrt{7}\left(20563200\sqrt{6}\right.\right.\\ & \left.\left.+8353800\right)-31187520\sqrt{5}+46721168\right)+u\left(\sqrt{7}\left(133660800\sqrt{6}+40098240\sqrt{5}+41769000\right)+100245600\sqrt{3}\right.\\ & \left.+218312640\sqrt{5}+\sqrt{2}\left(64774080\sqrt{5}+165110400\right)+922164490\right)-\sqrt{7}\left(20563200\sqrt{6}+8353800\right)+31187520\sqrt{5}\\ & \left.\left.\left.-46721168\right)/350859600\right)\right\} \end{array}$

5. $\begin{array}{cc} & HBM^{\#}\left(Marta-COF-1-BT\left(t,u\right)\right)=\left\{\left(457tu/33-457u^{2}/66-485t/792+953u/72-485/792\right),\right.\\ & \left(11993102286372172151tu/173128852216412400-11993102286372172151u^{2}/346257704432824800-37790603533t/5001138450\right.\\ & \left.\left.+57698305530932653121u/934895801968626960-37790603533/5001138450\right)\right\} \end{array}$

6. $\begin{array}{cc} & HTM^{\#}\left(Marta-COF-1-BT\left(t,u\right)\right.=\left\{\left(241633u/30780-10463t/30780+21008tu/2565-10504u^{2}/2565\right.\right.\\ & \left.-10463/30780\right),\left(205703218955993208339077tu/394830447428585376936000-205703218955993208339077u^{2}/789660894857170753872000\right.\\ & -55170047265049t/3645164474610750+1078528002248132799310459u/2132084416114361035454400\\ & \left.\left.-55170047265049/3645164474610750\right)\right\} \end{array}$

7. $\begin{array}{cc} & BMG^{\#}\left(Marta-COF-1-BT\left(t,u\right)\right)=\left\{\left(\left(tu\left(1056\sqrt{6}+3186\right)-u^{2}\left(528\sqrt{6}+1593\right)-t\left(88\sqrt{6}+81\right)\right.\right.\right.\\ & \left.\left.+u\left(968\sqrt{6}+3105\right)-88\sqrt{6}-81\right)/3\right),\left(\left(tu\left(13020\sqrt{3}+7308\sqrt{5}+\sqrt{2}\left(6678\sqrt{5}+37380\right)+\sqrt{7}\left(16500\sqrt{6}\right.\right.\right.\right.\\ & \left.\left.+3384\sqrt{5}+9480\right)+126630\right)-u^{2}\left(6510\sqrt{3}+3654\sqrt{5}+\sqrt{2}\left(3339\sqrt{5}+18690\right)+\sqrt{7}\left(8250\sqrt{6}+1692\sqrt{5}\right.\right.\\ & \left.\left.+4740\right)+63315\right)-t\left(\sqrt{7}\left(2200\sqrt{6}+1580\right)-1218\sqrt{5}+4515\right)+u\left(13020\sqrt{3}+8526\sqrt{5}+\sqrt{2}\left(6678\sqrt{5}\right.\right.\\ & \left.\left.\left.\left.\left.+37380\right)+\sqrt{7}\left(14300\sqrt{6}+3384\sqrt{5}+7900\right)+122115\right)+1218\sqrt{5}-\sqrt{7}\left(2200\sqrt{6}+1580\right)-4515\right)/105\right)\right\} \end{array}$

8. $\begin{array}{cc} & BMH^{\#}\left(Marta-COF-1-BT\left(t,u\right)\right)=\left\{13878tu-6939u^{2}-723t+13155u-723,200274tu-100137u^{2}\right.\\ & \left.-9849t+190425u-9849\right\} \end{array}$

9. $\begin{array}{cc} & TMG^{\#}\left(Marta-COF-1-BT\left(t,u\right)\right)=\left\{\left(\left(tu\left(1824\sqrt{6}+5562\right)-u^{2}\left(912\sqrt{6}+2781\right)-t\left(152\sqrt{6}+153\right)\right.\right.\right.\\ & \left.\left.+u\left(1672\sqrt{6}+5409\right)-152\sqrt{6}-153\right)/3\right),\left(\left(tu\left(31080\sqrt{3}+15372\sqrt{5}+\sqrt{2}\left(16254\sqrt{5}+91140\right)\right.\right.\right.\\ & \left.+\sqrt{7}\left(38100\sqrt{6}+7848\sqrt{5}+23160\right)+300510\right)-u^{2}\left(15540\sqrt{3}+7686\sqrt{5}+\sqrt{2}\left(8127\sqrt{5}+45570\right)\right.\\ & \left.+\sqrt{7}\left(19050\sqrt{6}+3924\sqrt{5}+11580\right)+150255\right)-t\left(\sqrt{7}\left(5080\sqrt{6}+3860\right)-2562\sqrt{5}+10395\right)+u\left(31080\sqrt{3}\right.\\ & \left.+17934\sqrt{5}+\sqrt{2}\left(16254\sqrt{5}+91140\right)+\sqrt{7}\left(33020\sqrt{6}+7848\sqrt{5}+19300\right)+290115\right)+2562\sqrt{5}\\ & \left.\left.\left.-\sqrt{7}\left(5080\sqrt{6}+3860\right)-10395\right)/105\right)\right\} \end{array}$

10. $\begin{array}{cc} & TMH^{\#}\left(Marta-COF-1-BT\left(t,u\right)\right)=\left\{24366tu-12183u^{2}-1279t+23087u-1279,478386tu\right.\\ & \left.-239193u^{2}-23281t+455105u-23281\right\} \end{array}$

11. $\begin{array}{cc} & BM^{\#}\left(Marta-COF-1-BT\left(t,u\right)\right)=\left\{5106tu-2553u^{2}-265t+4841u-265,20238tu-10119u^{2}\right.\\ & \left.-495t+19743u+3190\right\} \end{array}$

12. $\begin{array}{cc} & TM^{\#}\left(Marta-COF-1-BT\left(t,u\right)\right)=\left\{8922tu-4461u^{2}-469t+8453u-469,63750tu-31875u^{2}-3247t\right.\\ & \left.+60503u-3247\right\} \end{array}$

   The equations below generate the topological descriptors of Marta-COF-2-BT (t,u) .

$$\begin{array}{ll} \xi^{d}\left(Marta-COF-2-BT\left(t,u\right)\right) & =\left(4t+4u+4\right)\Gamma_{\text{BO}}\xi \left(2\Phi_{\text{O}},\Phi_{\text{B}}+\Phi_{\text{C}}\right)+\left(24tu-12u^{2}-4t+20u-4\right)\Gamma_{\text{BO}}\\ & \;\xi \left(2\Phi_{\text{O}}+\Phi_{\text{C}},\Phi_{\text{B}}+\Phi_{\text{C}}\right)+\left(12tu-6u^{2}-2t+10u-2\right)\Gamma_{\text{BC}}\xi \left(2\Phi_{\text{O}}+\Phi_{\text{C}},2\Phi_{\text{C}}+\Phi_{\text{B}}\right)\\ & \;+\left(24tu-12u^{2}+24u\right)\Gamma_{\text{OC}}\xi \left(\Phi_{\text{B}}+\Phi_{\text{C}},2\Phi_{\text{C}}+\Phi_{\text{O}}\right)+\left(120tu-60u^{2}+120u\right)\\ & \;\;\;\Gamma_{\text{CC}}\xi \left(3\Phi_{\text{C}},3\Phi_{\text{C}}\right)+\left(24tu-12u^{2}+24u\right)\Gamma_{\text{CC}}\xi \left(2\Phi_{\text{C}}+\Phi_{\text{O}},2\Phi_{\text{C}}\right)+\left(12tu-6u^{2}+12u\right)\\ & \;\;\;\Gamma_{\text{CC}}\xi \left(2\Phi_{\text{C}}+\Phi_{\text{O}},2\Phi_{\text{C}}+\Phi_{\text{O}}\right)+\left(72tu-36u^{2}-4t+68u-4\right)\Gamma_{\text{CC}}\\ & \;\;\;\xi \left(2\Phi_{\text{C}},3\Phi_{\text{C}}\right)+\left(48tu-24u^{2}-2t+46u-2\right)\Gamma_{\text{CC}}\xi \left(2\Phi_{\text{C}},2\Phi_{\text{C}}\right) \end{array}$$

$$\begin{array}{ll} \xi^{s}\left(Marta-COF-2-BT\left(t,u\right)\right) & =\left(4t+4u+4\right)\Gamma_{\text{BO}}\xi \left(4\Phi_{\text{O}},3\Phi_{\text{C}}+2\Phi_{\text{B}}\right)+\left(24tu-12u^{2}-4t+20u-4\right)\\ & \;\;\;\Gamma_{\text{BO}}\xi \left(4\Phi_{\text{O}}+3\Phi_{\text{C}},3\Phi_{\text{B}}+3\Phi_{\text{C}}\right)+\left(24tu-12u^{2}-4t+20u-4\right)\\ & \;\Gamma_{\text{OC}}\xi \left(3\Phi_{\text{B}}+3\Phi_{\text{C}},5\Phi_{\text{C}}+2\Phi_{\text{O}}\right)+\left(4t+4u+4\right)\Gamma_{\text{OC}}\xi \left(2\Phi_{\text{B}}+3\Phi_{\text{C}},5\Phi_{\text{C}}+2\Phi_{\text{O}}\right)\\ & \;+\left(12tu-6u^{2}+12u\right)\Gamma_{\text{CC}}\xi \left(2\Phi_{\text{O}}+5\Phi_{\text{C}},2\Phi_{\text{O}}+5\Phi_{\text{C}}\right)+\left(48tu-24u^{2}+48u\right)\\ & \;\;\;\Gamma_{\text{CC}}\xi \left(8\Phi_{\text{C}},9\Phi_{\text{C}}\right)+\left(24tu-12u^{2}+24u\right)\Gamma_{\text{CC}}\xi \left(8\Phi_{\text{C}},8\Phi_{\text{C}}\right)+\left(48tu-24u^{2}+48u\right)\\ & \;\;\;\Gamma_{\text{CC}}\xi \left(9\Phi_{\text{C}},9\Phi_{\text{C}}\right)+\left(24tu-12u^{2}+24u\right)\Gamma_{\text{CC}}\xi \left(6\Phi_{\text{C}},8\Phi_{\text{C}}\right)+\left(24tu-12u^{2}+24u\right)\\ & \;\;\;\Gamma_{\text{CC}}\xi \left(5\Phi_{\text{C}},8\Phi_{\text{C}}\right)+\left(24tu-12u^{2}+24u\right)\Gamma_{\text{CC}}\xi \left(4\Phi_{\text{C}},5\Phi_{\text{C}}\right)+\left(12tu-6u^{2}+12u\right)\\ & \;\;\;\Gamma_{\text{CC}}\xi \left(4\Phi_{\text{C}},4\Phi_{\text{C}}\right)+\left(24tu-12u^{2}+24u\right)\Gamma_{\text{CC}}\xi \left(6\Phi_{\text{C}},7\Phi_{\text{C}}\right)+\left(12tu-6u^{2}-2t+10u-2\right)\\ & \;\Gamma_{\text{CC}}\xi \left(5\Phi_{\text{C}},5\Phi_{\text{C}}\right)+\left(24tu-12u^{2}-4t+20u-4\right)\Gamma_{\text{CC}}\xi \left(5\Phi_{\text{C}},7\Phi_{\text{C}}\right)\\ & \;\;\;+\left(12tu-6u^{2}-2t+10u-2\right)\Gamma_{\text{BC}}\xi \left(4\Phi_{\text{O}}+3\Phi_{\text{C}},4\Phi_{\text{C}}+3\Phi_{\text{B}}\right). \end{array}$$

Result 2 The quantitative expressions for Marta-COF-2-BT (t,u) are given by

1. $\begin{array}{cc} & GH^{\#}\left(Marta-COF-2-BT\left(t,u\right)\right)=\left\{\left(2\left(tu\left(180\sqrt{6}+744\right)-u^{2}\left(90\sqrt{6}+372\right)-t\left(10\sqrt{6}+5\right)\right.\right.\right.\\ & \left.\left.+u\left(170\sqrt{6}+739\right)-10\sqrt{6}-5\right)\right),\left(2\left(tu\left(6120\sqrt{2}+1680\sqrt{3}+\sqrt{35}\left(234\sqrt{30}+360\right)+\sqrt{5}\left(780\sqrt{2}+540\right)\right.\right.\right.\\ & \left.+17730\right)-u^{2}\left(3060\sqrt{2}+840\sqrt{3}+\sqrt{35}\left(117\sqrt{30}+180\right)+\sqrt{5}\left(390\sqrt{2}+270\right)+8865\right)-t\left(130\sqrt{42}-90\sqrt{5}\right.\\ & \left.+370\right)+u\left(6120\sqrt{2}+1680\sqrt{3}+\sqrt{35}\left(208\sqrt{30}+360\right)+\sqrt{5}\left(780\sqrt{2}+630\right)+17360\right)+90\sqrt{5}\\ & \left.\left.\left.-130\sqrt{42}-370\right)/5\right)\right\} \end{array}$

2. $\begin{array}{cc} & GBM^{\#}\left(Marta-COF-2-BT\left(t,u\right)\right)=\left\{\left(\left(tu\left(1440\sqrt{6}+4488\right)-u^{2}\left(720\sqrt{6}+2244\right)\right.\right.\right.\\ & \left.\left.-t\left(80\sqrt{6}-11\right)+u\left(1360\sqrt{6}+4499\right)-80\sqrt{6}+11\right)/110\right),\left(\left(\left(tu\left(11173755146400\sqrt{2}+5346581763600\sqrt{3}\right.\right.\right.\right.\\ & \left.+\sqrt{35}\left(904058370936\sqrt{30}+1763234411400\right)+\sqrt{5}\left(3127245937200\sqrt{2}+5715311540400\right)+45388274429730\right)\\ & -u^{2}\left(\sqrt{35}\left(452029185468\sqrt{30}+881617205700\right)+\sqrt{5}\left(1563622968600\sqrt{2}+2857655770200\right)+5586877573200\sqrt{2}\right.\\ & \left.+2673290881800\sqrt{3}+22694137214865\right)-t\left(502254650520\sqrt{42}-952551923400\sqrt{5}+1753905128800\right)\\ & +u\left(\sqrt{35}\left(803607440832\sqrt{30}+1763234411400\right)+11173755146400\sqrt{2}+5346581763600\sqrt{3}+\sqrt{5}\left(3127245937200\sqrt{2}\right.\right.\\ & \left.\left.\left.\left.\left.+6667863463800\right)+43634369300930\right)+952551923400\sqrt{5}-502254650520\sqrt{42}-1753905128800\right)/3453000722325\right)\right\} \end{array}$

3. $\begin{array}{cc} & GTM^{\#}\left(Marta-COF-2-BT\left(t,u\right)\right)=\left\{\left(\left(tu\left(1296\sqrt{6}+4104\right)-u^{2}\left(648\sqrt{6}+2052\right)-t\left(72\sqrt{6}\right.\right.\right.\right.\\ & \left.\left.\left.-19\right)+u\left(1224\sqrt{6}+4123\right)-72\sqrt{6}+19\right)/171\right),\left(\left(tu\left(17411461829280\sqrt{2}+8509655894040\sqrt{3}\right.\right.\right.\\ & \left.+\sqrt{35}\left(1487514652344\sqrt{30}+2888598789720\right)+\sqrt{5}\left(4881508032240\sqrt{2}+10323189117360\right)+75049311782966\right)\\ & -u^{2}\left(\sqrt{35}\left(743757326172\sqrt{30}+1444299394860\right)+8705730914640\sqrt{2}+4254827947020\sqrt{3}\right.\\ & \left.+\sqrt{5}\left(2440754016120\sqrt{2}+5161594558680\right)+37524655891483\right)-t\left(826397029080\sqrt{42}-1720531519560\sqrt{5}\right.\\ & \left.+2998640648376\right)+u\left(\sqrt{35}\left(1322235246528\sqrt{30}+2888598789720\right)+17411461829280\sqrt{2}+8509655894040\sqrt{3}\right.\\ & \left.+\sqrt{5}\left(4881508032240\sqrt{2}+12043720636920\right)+72050671134590\right)+1720531519560\sqrt{5}-826397029080\sqrt{42}\\ & \left.\left.\left.-2998640648376\right)/13119052836645\right)\right\} \end{array}$

4. $\begin{array}{cc} & HG^{\#}\left(Marta-COF-2-BT\left(t,u\right)\right)=\left\{\left(\left(tu\left(864\sqrt{6}+2520\right)-u^{2}\left(432\sqrt{6}+1260\right)-t\left(48\sqrt{6}\right.\right.\right.\right.\\ & \left.\left.\left.-25\right)+u\left(816\sqrt{6}+2545\right)-48\sqrt{6}+25\right)/90\right),\left(\left(tu\left(55036800\sqrt{2}+33415200\sqrt{3}+\sqrt{35}\left(6168960\sqrt{30}+13366080\right)\right.\right.\right.\\ & \left.+\sqrt{5}\left(21591360\sqrt{2}+62375040\right)+314298686\right)-u^{2}\left(27518400\sqrt{2}+16707600\sqrt{3}+\sqrt{35}\left(3084480\sqrt{30}\right.\right.\\ & \left.\left.+6683040\right)+\sqrt{5}\left(10795680\sqrt{2}+31187520\right)+157149343\right)-t\left(3427200\sqrt{42}-10395840\sqrt{5}+14129856\right)\\ & +u\left(55036800\sqrt{2}+33415200\sqrt{3}+\sqrt{35}\left(5483520\sqrt{30}+13366080\right)+\sqrt{5}\left(21591360\sqrt{2}+72770880\right)\right.\\ & \left.\left.\left.\left.+300168830\right)+10395840\sqrt{5}-3427200\sqrt{42}-14129856\right)/116953200\right)\right\} \end{array}$

5. $\begin{array}{cc} & HBM^{\#}\left(Marta-COF-2-BT\left(t,u\right)\right)=\left\{\left(629tu/55-629u^{2}/110-833t/3960+8891u/792-833/3960\right),\right.\\ & \left(23267543517482263tu/23309638330633200-23267543517482263u^{2}/46619276661266400-351592t/45720675\right.\\ & \left.\left.+10158848653361669u/10256240865478608-351592/45720675\right)\right\} \end{array}$

6. $\begin{array}{cc} & HTM^{\#}\left(Marta-COF-2-BT\left(t,u\right)\right)=\left\{\left(5822tu/855-2911u^{2}/855-3379t/30780+206213u/30780\right.\right.\\ & \left.-3379/30780\right),\left(8185089994945153795501tu/18411782522576520168000-8185089994945153795501u^{2}/36823565045153040336000\right.\\ & \left.\left.-93117488t/38861857125+1628194648460053027721u/3682356504515304033600-93117488/38861857125\right)\right\} \end{array}$

7. $\begin{array}{cc} & BMG^{\#}\left(Marta-COF-2-BT\left(t,u\right)\right)=\left\{\left(2\left(tu\left(396\sqrt{6}+1368\right)-u^{2}\left(198\sqrt{6}+684\right)-t\left(22\sqrt{6}\right.\right.\right.\right.\\ & \left.\left.\left.+3\right)+u\left(374\sqrt{6}+1365\right)-22\sqrt{6}-3\right)/3\right),\left(\left(tu\left(37380\sqrt{2}+13020\sqrt{3}+\sqrt{35}\left(1980\sqrt{30}+3384\right)\right.\right.\right.\\ & \left.+\sqrt{5}\left(6678\sqrt{2}+7308\right)+119700\right)-u^{2}\left(18690\sqrt{2}+6510\sqrt{3}+\sqrt{35}\left(990\sqrt{30}+1692\right)+\sqrt{5}\left(3339\sqrt{2}+3654\right)\right.\\ & \left.+59850\right)-t\left(1100\sqrt{42}-1218\sqrt{5}+3360\right)+u\left(37380\sqrt{2}+13020\sqrt{3}+\sqrt{35}\left(1760\sqrt{30}+3384\right)\right.\\ & \left.\left.\left.\left.+\sqrt{5}\left(6678\sqrt{2}+8526\right)+116340\right)+1218\sqrt{5}-1100\sqrt{42}-3360\right)/105\right)\right\} \end{array}$

8. $\begin{array}{cc} & BMH^{\#}\left(Marta-COF-2-BT\left(t,u\right)\right)=\left\{\left(11208tu-5604u^{2}-278t+10930u-278\right),\left(162600tu\right.\right.\\ & \left.\left.-81300u^{2}-3570t+159030u-3570\right)\right\} \end{array}$

9. $\begin{array}{cc} & TMG^{\#}\left(Marta-COF-2-BT\left(t,u\right)\right)=\left\{\left(2\left(tu\left(684\sqrt{6}+2376\right)-u^{2}\left(342\sqrt{6}+1188\right)\right.\right.\right.\\ & \left.\left.-t\left(38\sqrt{6}+9\right)+u\left(646\sqrt{6}+2367\right)-38\sqrt{6}-9\right)/3\right),\left(\left(tu\left(91140\sqrt{2}+31080\sqrt{3}+\sqrt{35}\left(4572\sqrt{30}+7848\right)\right.\right.\right.\\ & \left.+\sqrt{5}\left(16254\sqrt{2}+15372\right)+283500\right)-u^{2}\left(45570\sqrt{2}+15540\sqrt{3}+\sqrt{35}\left(2286\sqrt{30}+3924\right)\right.\\ & \left.+\sqrt{5}\left(8127\sqrt{2}+7686\right)+141750\right)-t\left(2540\sqrt{42}-2562\sqrt{5}+7560\right)+u\left(91140\sqrt{2}+31080\sqrt{3}\right.\\ & \left.\left.\left.\left.+\sqrt{35}\left(4064\sqrt{30}+7848\right)+\sqrt{5}\left(16254\sqrt{2}+17934\right)+275940\right)+2562\sqrt{5}-2540\sqrt{42}-7560\right)/105\right)\right\} \end{array}$

10. $\begin{array}{cc} & TMH^{\#}\left(Marta-COF-2-BT\left(t,u\right)\right)=\left\{19656tu-9828u^{2}-494t+19162u-494,388584tu\right.\\ & \left.-194292u^{2}-8314t+380270u-8314\right\} \end{array}$

11. $\begin{array}{cc} & BM^{\#}\left(Marta-COF-2-BT\left(t,u\right)\right)=\left\{4128tu-2064u^{2}-102t+4026u-102,17748tu-8874u^{2}\right.\\ & \left.-80t+17668u+3605\right\} \end{array}$

12. $\begin{array}{cc} & TM^{\#}\left(Marta-COF-2-BT\left(t,u\right)\right)=\left\{7200tu-3600u^{2}-182t+7018u-182,51564tu-25782u^{2}\right.\\ & \left.-1216t+50348u-1216\right\} \end{array}$

     To determine entropy values for the two variations of Marta-COFs, we use the quantitative expressions from the above derived results with the aid of scalar multiplicative self-powered descriptors. Let $D_{1}=\left\{\left(2,2\right),\left(2,3\right),\left(3,3\right)\right\}$ and $S_{1}=\left\{\left(4,4\right),\left(4,5\right),\left(5,5\right),\left(5,7\right),\left(5,8\right),\left(6,7\right),\left(6,8\right),\left(7,7\right),\left(7,9\right),\left(8,8\right),\left(8,9\right),\left(9,9\right)\right\}$ . We denote $\xi_{\alpha_{1}}=\prod_{\left(r,x\right)\in D_{1}}{\xi \left(r,x\right)}^{\xi \left(r,x\right)}$ and $\xi_{\beta_{1}}=\prod_{\left(r,x\right)\in S_{1}}{\xi \left(r,x\right)}^{\xi \left(r,x\right)}$ . Thus, the mathematical expressions representing Marta-COF-1 as self-powered descriptors are provided below.

1. $\begin{array}{cc} & \xi^{dp^{*}}\left(\text{Marta}-\text{COF}-1-\text{BT}\left(t,u\right)\right)=\xi_{\alpha_{1}}\left(\left(48tu-24u^{2}+2t+50u+2\right)\left(-174tu+87u^{2}\right.\right.\\ & \left.\left.+7t-167u+7\right)\left(-192tu+96u^{2}+16t-176u+16\right)\right) \end{array}$

2. $\begin{array}{cc} & \xi^{sp^{*}}\left(\text{Marta}-\text{COF}-1-\text{BT}\left(t,u\right)\right)=\xi_{\beta_{1}}\left(-2985984u^{6}{\left(2t-u+2\right)}^{6}\left(-54tu+27u^{2}\right.\right.\\ & \left.+t-53u+1\right)\left(-12tu+6u^{2}+2t-10u+2\right)\left(-24tu+12u^{2}+2t-22u+2\right)\left(-24tu+12u^{2}+4t-20u+4\right)\left(24tu-12u^{2}+4t+28u+4\right)\\ & \left.\left(-120tu+60u^{2}+16t-104u+16\right)\right) \end{array}$

Similarly for Marta-COF-2-BT (t,u) , let $D_{2}=\left\{\left(2,2\right),\left(2,3\right),\left(3,3\right)\right\}$ and $S_{2}=\left\{\left(4,4\right),\left(4,5\right),\left(5,5\right),\left(5,7\right),\left(5,8\right),\left(6,7\right),\left(6,8\right),\left(7,7\right),\left(8,8\right),\left(8,9\right),\left(9,9\right)\right\}$ . We denote $\xi_{\alpha_{2}}=\prod_{\left(r,x\right)\in D_{2}}{\xi \left(r,x\right)}^{\xi \left(r,x\right)}$ and $\xi_{\beta_{2}}=\prod_{\left(r,x\right)\in S_{2}}{\xi \left(r,x\right)}^{\xi \left(r,x\right)}$ . Then,

1. $\begin{array}{cc} & \xi^{dp^{*}}\left(\text{Marta}-\text{COF}-2-\text{BT}\left(t,u\right)\right)=\xi_{\alpha_{2}}\left(\left(48tu-24u^{2}+2t+50u+2\right)\left(-144tu+72u^{2}\right.\right.\\ & \left.\left.+2t-142u+2\right)\left(-144tu+72u^{2}+8t-136u+8\right)\right) \end{array}$

2. $\begin{array}{cc} & \xi^{sp^{*}}\left(\text{Marta}-\text{COF}-2-\text{BT}\left(t,u\right)\right)=\xi_{\beta_{2}}\left(-71663616u^{7}{\left(2t-u+2\right)}^{7}\left(-12tu+6u^{2}\right.\right.\\ & \left.\left.+2t-10u+2\right)\left(-24tu+12u^{2}+2t-22u+2\right)\left(24tu-12u^{2}+4t+28u+4\right)\left(-72tu+36u^{2}+8t-64u+8\right)\right) \end{array}$

We are now ready to calculate the entropies of Marta-COFs using the provided mathematical expressions. Due to the complexity of these expressions, we determine the numerical values of Marta-COFs where the dimensions of the bi-trapezium configuration are set by BT (t,t) . The computed entropies are presented in Tables 3, 4. Comparing the various descriptors, the tri-Zagreb-harmonic consistently demonstrates higher entropy values across all configuration phases in both Marta-COFs.

**FIGURE 5:**
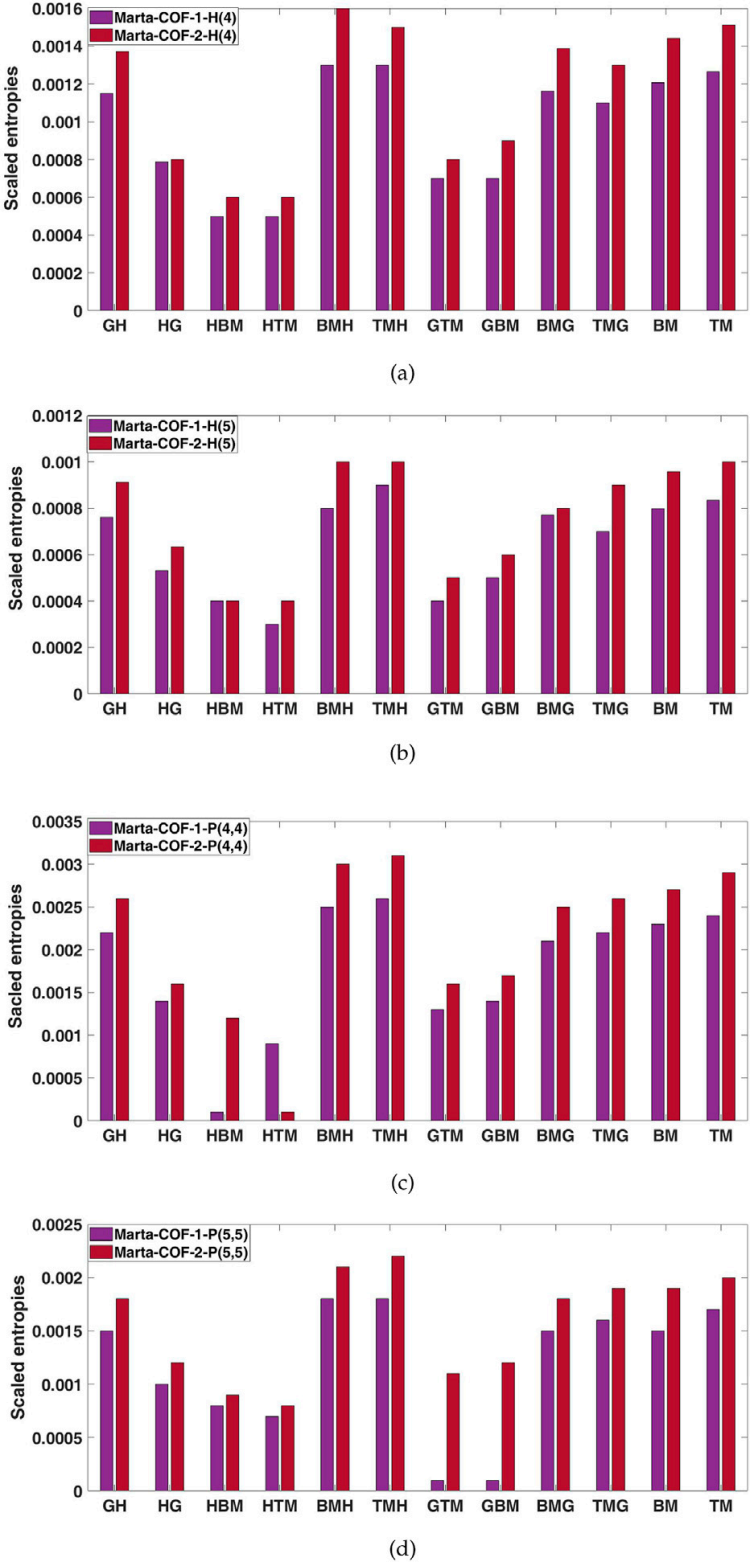
Bar diagrams of scaled entropies **(A, B)** Marta-COF-1-H (u) and Marta-COF-2-H (u) , **(C, D)** Marta-COF-1-P (t,t) and Marta-COF-2-P. (t,t) .

The entropies calculated for Marta-COF-1 and Marta-COF-2 primarily depend on their total number of bonds, which is unequal due to the fixed dimensions of these COFs. To compare their entropies effectively and investigate structural characteristics like bond energy and stability, we employ a scaling process. We perform scaling for the hexagonal and parallelogram configurations of Marta-COFs between two variations by calculating the ratio of total degree entropies to the total number of bonds. Table 5 clearly shows that the bond-wise entropies of the Marta-COF-2 framework are consistently higher than those of Marta-COF-1 across all hexagonal and parallelogram configurations, as depicted in Figure 5. As a result, the Marta-COF-2 frameworks exhibit a higher degree of information disorder than the Marta-COF-1 frameworks.

**TABLE 3:** Entropies calculated from degree/degree-sum parameters of Marta-COF-1-BT (t,t) .

ξ	d	t=2	t=3	t=4	t=5	t=6	t=7	t=8	t=9	t=10
	s									
GH		9.297	9.946	10.427	10.811	11.132	11.408	11.650	11.865	12.059
		11.249	11.908	12.393	12.780	13.102	13.379	13.621	13.837	14.031
GBM		5.765	6.432	6.921	7.311	7.634	7.912	8.155	8.371	8.566
		4.877	5.641	6.178	6.594	6.935	7.224	7.476	7.698	7.898
GTM		5.187	5.869	6.366	6.759	7.085	7.364	7.609	7.826	8.021
		3.628	4.555	5.171	5.633	6.002	6.310	6.575	6.808	7.015
HG		5.391	6.066	6.559	6.950	7.275	7.553	7.797	8.014	8.209
		2.095	3.337	4.106	4.654	5.078	5.423	5.714	5.966	6.187
HBM		3.612	4.400	4.9460	5.366	5.709	5.999	6.251	6.474	6.674
		5.261	6.018	6.552	6.967	7.308	7.597	7.849	8.072	8.272
HTM		2.876	3.751	4.338	4.782	5.139	5.439	5.698	5.926	6.129
		−25.217	−14.384	−8.897	−5.637	−3.495	−1.986	−0.866	−0.002	0.685
BMG		8.876	9.525	10.006	10.390	10.711	10.986	11.228	11.443	11.638
		9.523	10.178	10.662	11.048	11.370	11.646	11.888	12.104	12.298
BMH		10.851	11.499	11.981	12.365	12.686	12.962	13.203	13.419	13.613
		13.487	14.152	14.640	15.028	15.351	15.628	15.871	16.087	16.281
TMG		9.428	10.076	10.557	10.942	11.263	11.538	11.780	11.995	12.190
		10.377	11.034	11.519	11.905	12.227	12.504	12.746	12.962	13.156
TMH		11.412	12.062	12.543	12.928	13.249	13.524	13.766	13.982	14.176
		14.352	15.021	15.510	15.898	16.221	16.498	16.741	16.957	17.152
BM		9.852	10.501	10.981	11.366	11.686	11.962	12.204	12.419	12.613
		11.279	11.911	12.384	12.763	13.081	13.354	13.594	13.808	14.001
TM		10.408	11.057	11.539	11.923	12.244	12.520	12.762	12.977	13.171
		12.347	12.464	13.497	13.884	14.207	14.483	14.726	14.942	15.136

**TABLE 4:** Entropies calculated from degree/degree-sum parameters of Marta-COF-2-BT (t,t) .

ξ	d	t=2	t=3	t=4	t=5	t=6	t=7	t=8	t=9	t=10
	s									
GH		9.119	9.758	10.234	10.615	10.934	11.207	11.448	11.662	11.855
		11.071	11.721	12.202	12.585	12.905	13.180	13.421	13.635	13.829
GBM		5.585	6.246	6.732	7.119	7.441	7.717	7.959	8.174	8.369
		4.686	5.449	5.985	6.401	6.741	7.030	7.281	7.503	7.702
GTM		5.006	5.684	6.178	6.570	6.895	7.173	7.416	7.632	7.827
		3.411	4.346	4.967	5.431	5.803	6.112	6.378	6.611	6.818
HG		5.218	5.888	6.378	6.767	7.091	7.367	7.611	7.826	8.021
		1.860	3.121	3.901	4.455	4.884	5.232	5.526	5.779	6.002
HBM		3.404	4.202	4.752	5.175	5.519	5.811	6.063	6.286	6.486
		−10.573	−5.293	−2.516	−0.805	0.357	1.205	1.854	2.371	2.796
HTM		2.648	3.543	4.139	4.588	4.949	5.251	5.511	5.740	5.945
		−26.290	−15.212	−9.558	−6.185	−3.965	−2.399	−1.237	−0.341	0.372
BMG		8.701	9.340	9.815	10.196	10.515	10.789	11.029	11.243	11.436
		9.345	9.991	10.471	10.853	11.172	11.447	11.688	11.902	12.096
BMH		10.671	11.311	11.788	12.169	12.487	12.761	13.001	13.216	13.409
		13.312	13.969	14.452	14.837	15.157	15.432	15.674	15.889	16.082
TMG		9.249	9.889	10.365	10.746	11.064	11.338	11.578	11.793	11.986
		10.198	10.847	11.327	11.710	12.029	12.304	12.545	12.759	12.953
TMH		11.232	11.872	12.349	12.731	13.049	13.323	13.563	13.777	13.971
		14.178	14.838	15.322	15.707	16.028	16.303	16.545	16.759	16.953
BM		9.674	10.313	10.789	11.171	11.489	11.762	12.003	12.217	12.411
		11.179	11.802	12.270	12.646	12.961	13.233	13.472	13.685	13.877
TM		10.229	10.869	11.345	11.726	12.044	12.318	12.559	12.773	12.966
		12.169	12.823	13.305	13.689	14.009	14.284	14.525	14.740	14.934

**TABLE 5:** Scaled entropy values for parallelogram and hexagonal configurations between Marta-COF-1 and Marta-COF-2.

$\xi^{d}$	t=u=4		t=u=5		t=7,u=4		t=9,u=5	
	Marta-COF-1	Marta-COF-2	Marta-COF-1	Marta-COF-2	Marta-COF-1	Marta-COF-2	Marta-COF-1	Marta-COF-2
GH	0.0022	0.0026	0.0015	0.0018	0.0011	0.0013	0.0007	0.0009
HG	0.0014	0.0016	0.001	0.0012	0.0007	0.0008	0.0005	0.0006
HBM	0.001	0.0012	0.0008	0.0009	0.0005	0.0006	0.0004	0.0004
HTM	0.0009	0.001	0.0007	0.0008	0.0005	0.0006	0.0003	0.0004
BMH	0.0025	0.0030	0.0018	0.0021	0.0013	0.0015	0.0008	0.0010
TMH	0.0026	0.0031	0.0018	0.0022	0.0013	0.0016	0.0009	0.0010
GTM	0.0013	0.0016	0.001	0.0011	0.0007	0.0008	0.0004	0.0005
GBM	0.0014	0.0017	0.001	0.0012	0.0007	0.0009	0.0005	0.0006
BMG	0.0021	0.0025	0.0015	0.0018	0.0011	0.0013	0.0007	0.0008
TMG	0.0022	0.0026	0.0016	0.0019	0.0011	0.0013	0.0007	0.0009
BM	0.0023	0.0027	0.0016	0.0019	0.0012	0.0014	0.0007	0.0009
TM	0.0024	0.0029	0.0017	0.0020	0.0012	0.0015	0.0008	0.0010

## 4 Prediction of graph energy

A prominent application of spectral graph theory is its ability to relate graph spectrum to the molecular orbital energy levels of π -electrons in conjugated hydrocarbons (Graovac et al., 1975; Gutman and Furtula, 2017). The concept of total π -electron energy originated from Hückel molecular orbital theory, specifically for alternant hydrocarbons frameworks. In spectral graph theory, the π -electron energy is approximately proportional to the graph-based energy for alternant hydrocarbons; however, this does not hold for general frameworks. Nevertheless, this approach can be extended to graphs containing heteroatoms by treating them similarly to graphs composed of carbon atoms. Let G be a graph of order n with adjacency matrix A . The eigenvalues of A are denoted as $\lambda_{1},\lambda_{2},\lambda_{3}$ , …, $\lambda_{n}$ constitute graph spectrum (Gutman, 1978; Kalaam et al., 2024). The graph energy $E_{\pi }\left(G\right)$ , typically expressed in β -units, for a graph G is defined as the sum of the absolute values of its eigenvalues, as shown below.

$$E_{\pi }\left(G\right)=\overset{n}{\underset{i=1}{\sum }}|\lambda_{i}|$$

Evaluating the graph energy of Marta covalent organic frameworks in higher-order dimensions (t,u) presents challenges in generating adjacency matrices and solving the associated problem. However, software like newGRAPH (Stevanović et al., 2021) is useful to some extent for addressing this issue in smaller-dimensional frameworks. Therefore, we compute the energy values for specific graph frameworks of (t,u) using the newGRAPH software, as shown in Table 6. Based on these values, we developed statistical models to predict the energy values for higher dimensions by consolidating data from various frameworks into a unified dataset.

**TABLE 6:** Energy values for Marta-COF-1-BT (t,u) and Marta-COF-2-BT (t,u) .

Marta-COF-1-BT (t,u)	$E_{\pi }$ in β units	Marta-COF-2-BT (t,u)	$E_{\pi }$ in β units
(1,1)	629.91736	(1,1)	542.93298
(2,1)	1073.14678	(2,1)	913.67542
(2,2)	1749.63317	(2,2)	1474.18263
(3,1)	1516.37621	(3,1)	1284.41785
(3,2)	2659.37652	(3,2)	2224.45461
(3,3)	3335.86291	(3,3)	2784.96182
(4,1)	1959.60563	(4,1)	1655.16029
(4,2)	3569.11987	(4,2)	2974.72660

We conducted a correlation analysis to explore the relationship between topological descriptors and graph energy in two Marta-COFs. Next, we applied simple linear regression to examine the relationship between these two quantitative variables, providing a clear representation of the link between the predictor and the dependent variable. The proposed equation relating graph energy to topological descriptors is presented below.

$$E_{\pi }\left(G\right)=s\;\left(\xi \right)+c$$

where s and c are constants, and we also include the other statistical parameters such as standard error (Se) and the F -value.

Based on the correlation analysis, we identified the optimal predictive models for Marta-COF-1 and Marta-COF-2 based on degree descriptors. The geometric-bi-Zagreb index yielded a perfect correlation for both frameworks, with the lowest standard error (Se) and the highest F value. The linear regression equations derived from the geometric-bi-Zagreb index are presented below.

$$\begin{array}{ll} E_{\pi }\left(Marta-COF-1-BT\left(t,u\right)\right) & =5.209017\left(GBM^{d}\right)-0.0981429,\\ & \;r=1,\;F=32311498295.2312,\;Se=0.015422545959363\\ E_{\pi }\left(Marta-COF-2-BT\left(t,u\right)\right) & =5.208355\left(GBM^{d}\right)-0.077795,\\ & \;r=1,\;F=35031486119.8121,\;Se=0.0122440986858545 \end{array}$$

In the same way, the linear regression equations derived from degree-sum descriptors particularly the bi-Zagreb harmonic index for Marta-COF-1-BT (t,u) and the tri-Zagreb index for Marta-COF-2-BT (t,u) yield the most accurate predictive models, as shown below.

$$\begin{array}{ll} E_{\pi }\left(Marta-COF-1-BT\left(t,u\right)\right) & =0.00232835\left(BMH^{s}\right)-0.929803,\\ & \;r=0.999999991662206,\;F=359807408.29467,\;Se=0.146150251635939\\ E_{\pi }\left(Marta-COF-2-BT\left(t,u\right)\right) & =0.007362\left(TM^{s}\right)+0.435501,\\ & \;r=0.999999998716673,\;F=1117831165.98009,\;Se=0.0685437710167504 \end{array}$$

**FIGURE 6:**
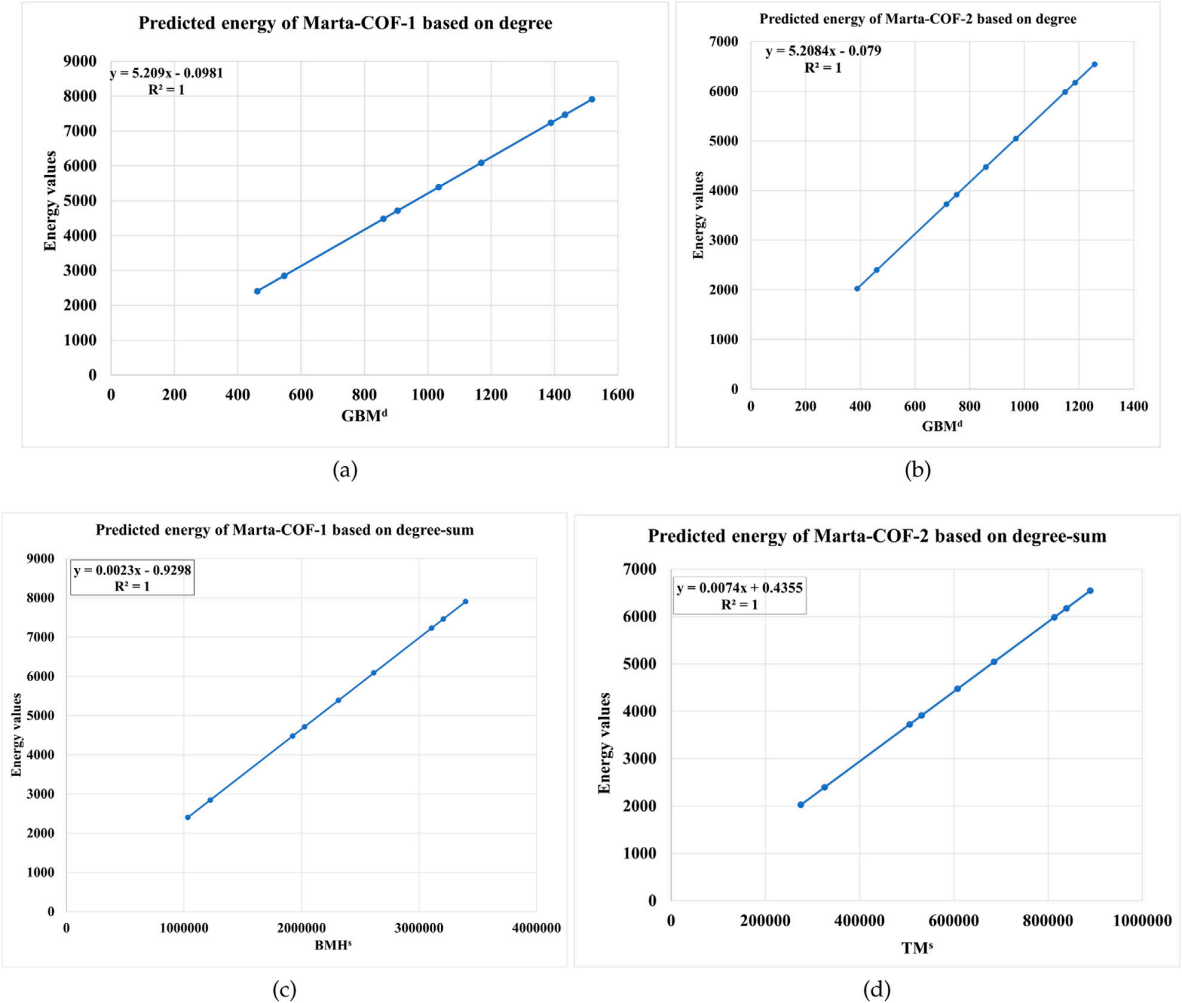
Comparison of predicted graph energy based on degree/degree-sum **(A, B)** Marta-COF-1-BT (t,u) and **(C, D)** Marta-COF-2-BT. (t,u) .

Using the regression equations mentioned above, we estimated the graph energy of Marta-COF-1 and Marta-COF-2 based on both degree and degree-sum descriptors in higher dimensions. The resulting predictions are presented in Tables 7, 8 and visually depicted in Figure 6. The predicted energy of Marta-COFs based on degree descriptors shows a perfect correlation compared to degree-sum descriptors, making these predictive models useful for estimating graph energy values in higher-dimensional Marta-COFs.

**TABLE 7:** Comparison of predicted energy for Marta-COF-1 based on degree and degree-sum descriptors.

Marta-COF-1-BT (t,u)	Predicted $E_{\pi }$ in β units by $GBM^{d}$	Predicted $E_{\pi }$ in β units by $BMH^{s}$
Marta-COF-1-BT (4,3)	4712.088025	4711.848507
Marta-COF-1-BT (4,4)	5388.629944	5388.378539
Marta-COF-1-BT (5,1)	2402.877657	2403.240586
Marta-COF-1-BT (5,2)	4478.844391	4478.694523
Marta-COF-1-BT (5,3)	6088.200927	6087.840491
Marta-COF-1-BT (5,4)	7231.059257	7230.678492
Marta-COF-1-BT (5,5)	7907.710565	7907.208525
Marta-COF-1-BT (6,1)	2846.122289	2846.616635
Marta-COF-1-BT (6,2)	5388.629944	5388.378539
Marta-COF-1-BT (6,3)	7464.423218	7463.832476

**TABLE 8:** Comparison of predicted energy for Marta-COF-2 based on degree and degree-sum descriptors.

Marta-COF-2-BT (t,u)	Predicted $E_{\pi }$ in β units by $GBM^{d}$	Predicted $E_{\pi }$ in β units by $TM^{s}$
Marta-COF-2-BT (4,3)	3914.740053	3914.766728
Marta-COF-2-BT (4,4)	4475.250718	4475.235789
Marta-COF-2-BT (5,1)	2025.936362	2025.648081
Marta-COF-2-BT (5,2)	3724.984055	3724.959645
Marta-COF-2-BT (5,3)	5044.519232	5044.657041
Marta-COF-2-BT (5,4)	5984.3221	5984.740269
Marta-COF-2-BT (5,5)	6545.261934	6545.209329
Marta-COF-2-BT (6,1)	2396.688729	2396.310057
Marta-COF-2-BT (6,2)	4475.250718	4475.235789
Marta-COF-2-BT (6,3)	6174.427058	6174.547353

## 5 Conclusion

The mathematical expressions for topological descriptors have been formulated, and entropy quantities for two variations of Marta-COFs have been derived. A refined edge partition technique has been employed, involving the use of innovative hybrid descriptors that combine geometric, harmonic, and Zagreb descriptors. Furthermore, a comparative analysis between Marta-COF-1 and Marta-COF-2 has been conducted, revealing that higher entropy values were consistently displayed by Marta-COF-2 in both hexagonal and parallelogram frameworks compared to Marta-COF-1. Optimal linear regression models to predict graph energy across different dimensional Marta frameworks have also been developed, significantly reducing computational complexity. These findings and techniques can be applied to link properties such as mechanical stability, solubility, hardness, and electrophilicity, provided that experimental data are available.

## Data Availability

The original contributions presented in the study are included in the article/supplementary material, further inquiries can be directed to the corresponding author.
